# Supplementary material for: RAB13 mRNA compartmentalisation spatially orients tissue morphogenesis
Source: EMBO J. 2020 Sep 18;39(21):e106003. doi: 10.15252/embj.2020106003 (PMC7604621; doi:10.15252/embj.2020106003)
Supplement: Supplementary file 1 — Expanded View Figures PDF [file EMBJ-39-e106003-s001.pdf]

Expanded View Figures

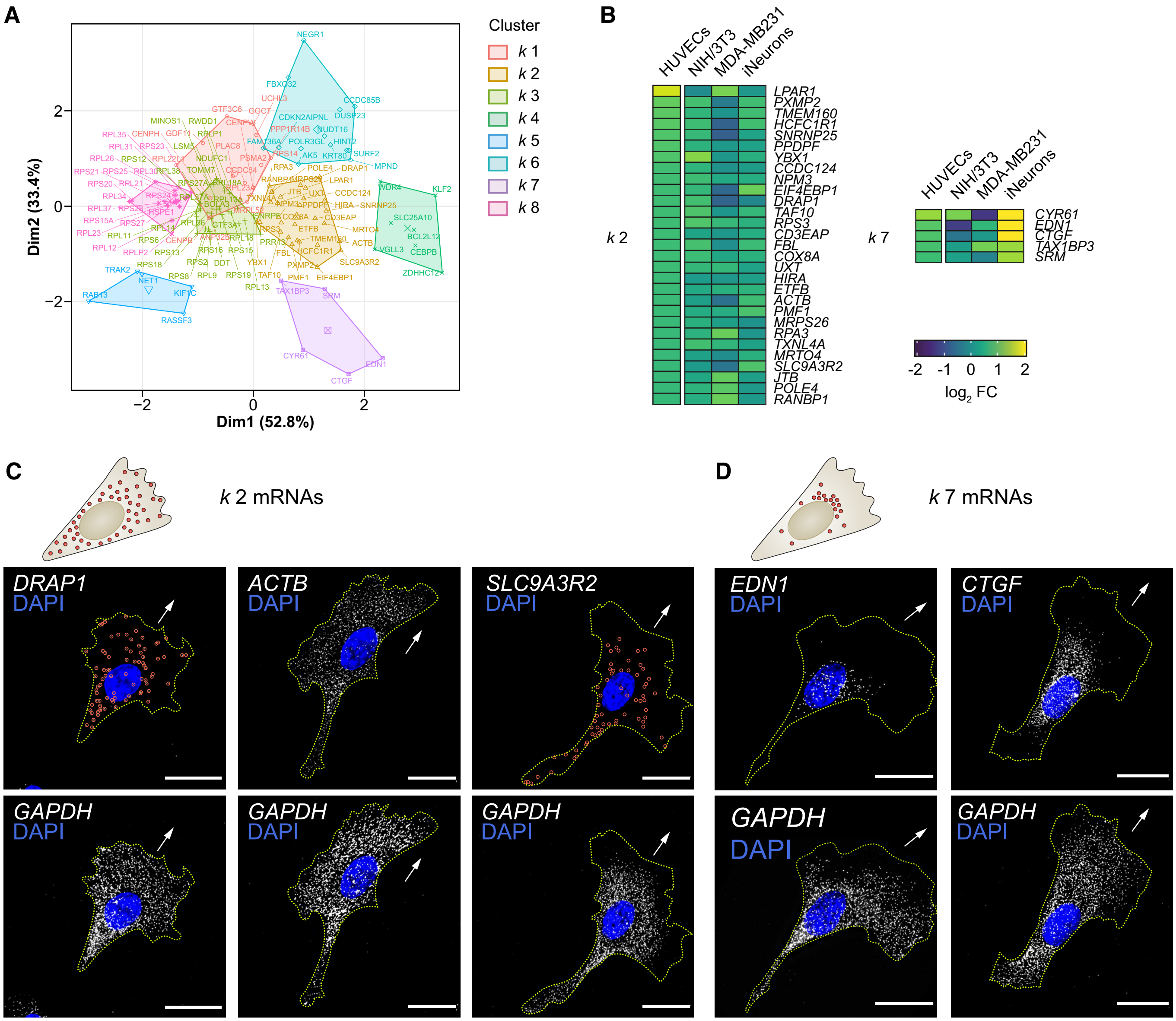

Figure EV1. Clustering of RNAseq datasets defines unexpected cell type-specific diversity to mRNA polarisation.

A Principal component plot depicting the *k*-means clustering analysis of mRNAs enriched across cell protrusion types.

B Detail of the heat map shown in Fig 1C representing $\log_2$ fold change (FC) levels (protrusions over cell bodies) of mRNAs present in clusters *k* 2 and *k* 7. The corresponding HUVEC $\log_2$ FC levels are shown in parallel.

C Top: distribution pattern of mRNAs clustered in *k* 2. Bottom: smFISH co-detection of *k* 2 mRNAs and *GAPDH* in HUVECs.

D Top: distribution pattern of mRNAs clustered in *k* 7. Bottom: smFISH co-detection of *k* 7 mRNAs and *GAPDH* in HUVECs.

Data information: arrows indicate the orientation of RNA localisation; yellow dashed lines outline cell borders; red circles highlight smFISH spots; scale bars = 20 μm (C, D).

© 2020 The Authors

A

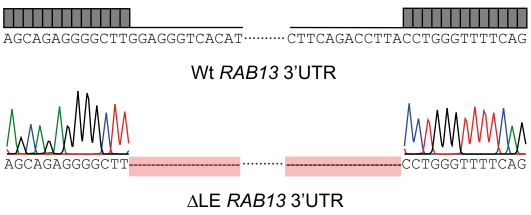

C

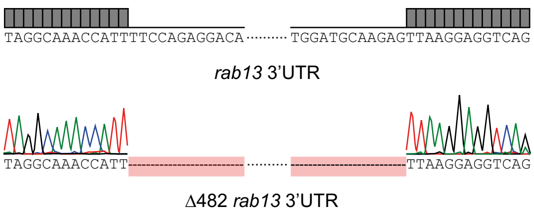

B

gRNA	Off-target locus	Gene	Region	Notes
5'	chr11:-134379673	*B3GAT1*	3'UTR	Not expressed
	chr4:-177812822	*LINC01099*	3'UTR	Not expressed
	chr5:-72194126	*MAP1B*	CDS	**No mismatches**
	chr17:+17167885	*MPRIP*	CDS	**No mismatches**
	chr1:+151527003	*CGN*	CDS	Not expressed
	chr11:-134379673	*B3GAT1*	3'UTR	Not expressed
3'	chr9:+130903598	*FIBCD1*	3'UTR	Not expressed
	chr19:-23526428	*LOC105372337*	3'UTR	Not expressed
	chr16:+23670135	*DCTN5*	3'UTR	**No mismatches**
	chr2:+85059287	*KCMF1*	3'UTR	**No mismatches**
	chr9:+104776159	*NIPSNAP3B*	3'UTR	Not expressed
	chr3:+133766306	*TF*	CDS	Not expressed
	chrX:+147271017	*LOC105373347*	3'UTR	Not expressed

D

gRNA	Off-target locus	Gene	Region	Notes
5'	chr13:-31783248	*hif1aa*	CDS	**No mismatches**
	chr9:+47436246	*tnsb1*	3'UTR	**No mismatches**
	chr25:+34666114	*cabz01080568.1*	CDS	**No mismatches**
	chr19:-10383593	*zgc:194578*	CDS	**No mismatches**
	chr16:+46997410	*thsd7ab*	CDS	Not expressed
	chr5:-60687396	*tmem132e*	3'UTR	**No mismatches**
3'	chr24:+24064786	*zgc:194578*	CDS	Not expressed
	chr16:-20831666	*tax1bp1b*	CDS	**No mismatches**
	chr6:+21514803	*cacng4a*	3'UTR	**No mismatches**

Figure EV2. CRISPR-Cas9 editing of the *RAB* 3′UTR *in vitro and in vivo* does not generate off-target mutations.

A Chromatogram confirming the excision of the LE within *RAB13* 3′UTR in HUVECs.
B List of predicted CRISPR-Cas9 off-target genes and RNAseq mismatch detection in CRISPR-Cas9-derived HUVEC clones (n = 1 each genotype).
C Chromatogram confirming the CRISPR-Cas9-mediated excision of 482-nt within the *rab13* 3′UTR in zebrafish embryos.
D List of predicted CRISPR-Cas9 off-target genes and RNAseq mismatch detection in *Tg*(*kdrl:EGFP*) *rab13*$^{+/+}$ and *rab13*$^{\Delta 3'UTR/\Delta 3'UTR}$ embryos (n = 2 each genotype).

© 2020 The Authors

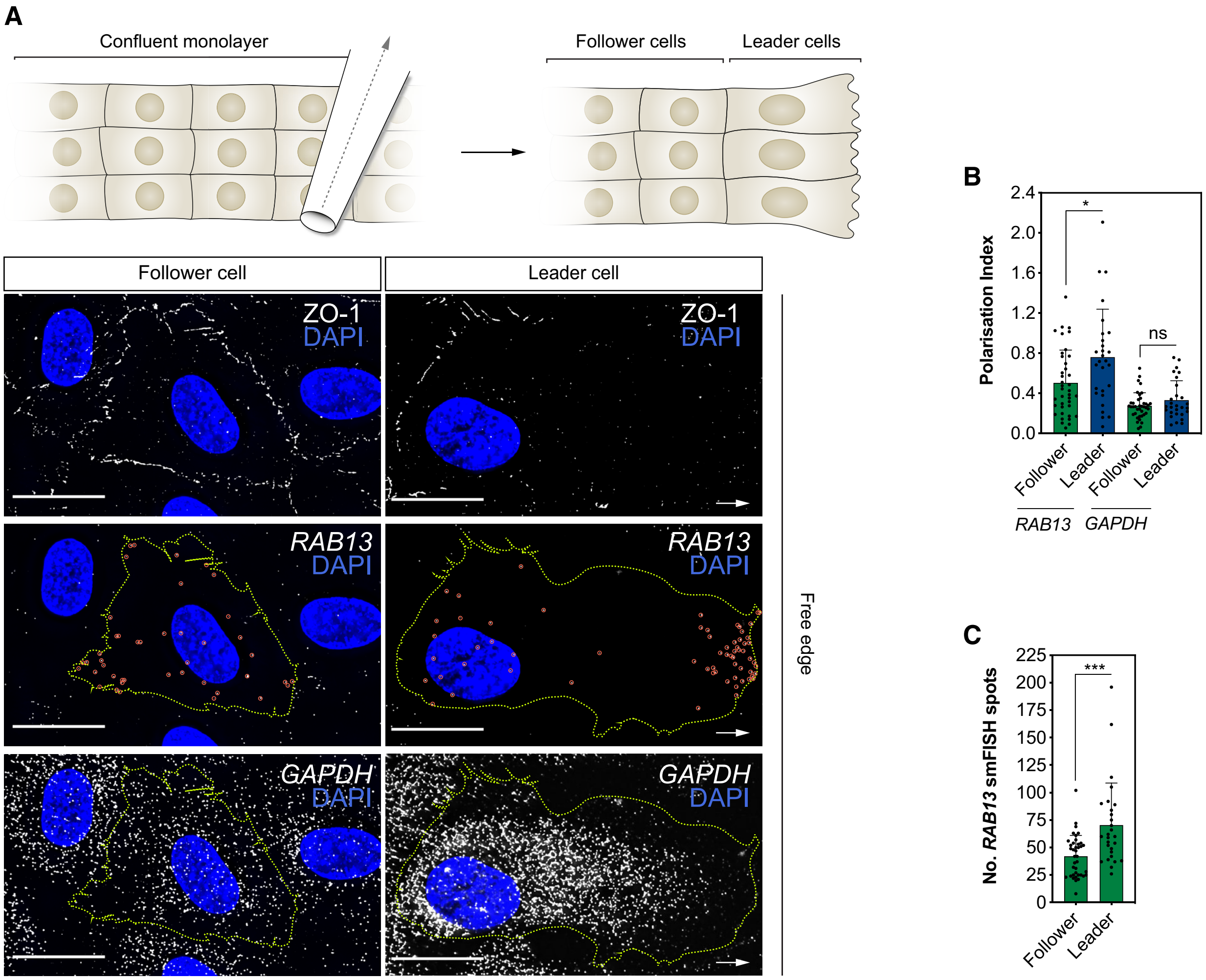

Figure EV3. Induction of endothelial cell collective migration drives *RAB13* mRNA polarisation in leader cells.

A Top: scratch wound assay generates a free edge on a confluent monolayer of HUVECs and encourages cell migration. Bottom: smFISH co-detection of *RAB13* mRNA and *GAPDH* mRNA in representative HUVECs migrating in a scratch wound assay. ZO-1 immunolabelling defines cell boundaries.

B Polarisation Index of *RAB13* and *GAPDH* co-detected in HUVECs cultured in scratch wound assays ($n \geq 28$ cells; *$P < 0.05$, ns: not significant; Mann–Whitney test).

C Quantification of the number of *RAB13* mRNA smFISH spots per cell ($n \geq 28$ cells; ***$P < 0.001$; Mann–Whitney test). Leader: cells identified at the edge of the scratch; follower: cells identified in confluent regions adjacent to leader cells.

Data information: arrows indicate orientation of RNA localisation; yellow dashed lines outline cell borders; red circles highlight smFISH spots; scale bars = 20 μm (A). Bar charts are presented as means ± s.d.

© 2020 The Authors

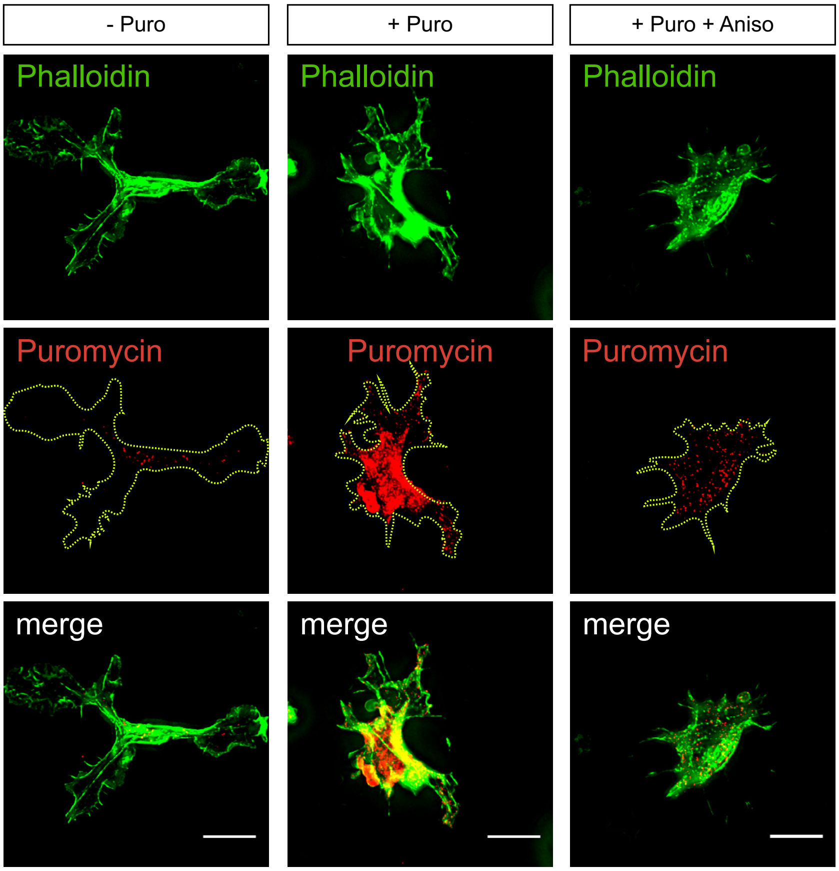

Figure EV4. Protein translation in endothelial cell protrusions.

Immunofluorescence analysis of HUVEC protrusions generated on the underside of Transwell membranes and exposed to puromycin after cell body removal. Yellow dashed lines outline protrusion borders; scale bars = 10 µm.

© 2020 The Authors

A

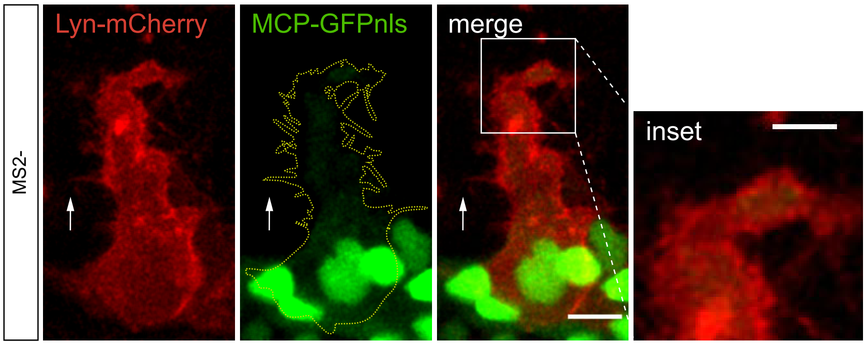

B

rab13 3'UTR conservation (%)

	Zebrafish	Mouse	Macaque	Human
**Zebrafish**	100.00	50.99	54.85	56.68
**Mouse**	50.99	100.00	71.99	71.86
**Macaque**	54.85	71.99	100.00	94.90
**Human**	56.68	71.86	94.90	100.00

C

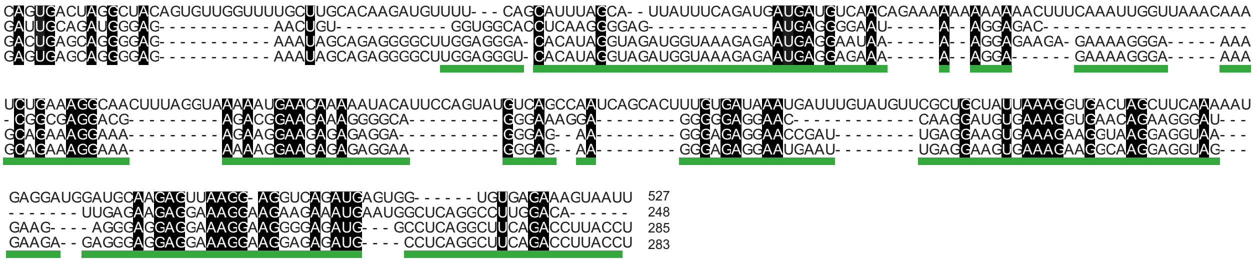

D

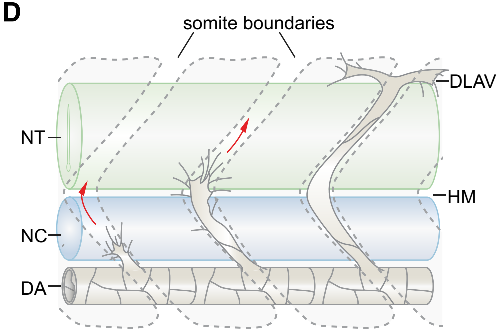

Figure EV5. The 3′UTR of *rab13* shows low conservation across species whilst retaining mRNA localisation potential.

A *Tg(fli1ep:MCP-GFPnls)* tip cell expressing a control Lyn-mCherry-24xMS2 construct.

B Percentage identity matrix of *rab13* 3′UTR orthologue sequences.

C Multiple sequence alignment between *rab13* 3′UTR orthologues. Black boxes indicate absolute nucleotide similarity. The human *RAB13* 3′UTR localisation element is underlined in green.

D Scheme depicts stages of zebrafish ISV sprouting. DA: dorsal aorta; DLAV: dorsal longitudinal anastomotic vessel; HM: horizontal myoseptum; NC: notochord; NT: neural tube.

Data information: white arrows indicate direction of ISV sprouting; yellow dashed line outlines ISV cell borders; scale bars = 10 μm; scale bar in inset = 5 μm (A).

© 2020 The Authors
