# Supplementary material for: RAB13 mRNA compartmentalisation spatially orients tissue morphogenesis
Source: EMBO J. 2020 Sep 18;39(21):e106003. doi: 10.15252/embj.2020106003 (PMC7604621; doi:10.15252/embj.2020106003)
Supplement: Supplementary file 7 — Movie EV1 [file EMBJ-39-e106003-s007.zip › 106003_Extended View Movies EV1/106003_Extended View Movie EV1 Legend.docx]

**Movie EV1.** Confocal time-lapse imaging of a representative endothelial cell co-transfected with plasmids expressing Lyn-mCherry, MCP-GFPnls and 24xMS2-*RAB13* 3’UTR. Arrowheads represent newly-generated filopodia.
