# Supplementary material for: RAB13 mRNA compartmentalisation spatially orients tissue morphogenesis
Source: EMBO J. 2020 Sep 18;39(21):e106003. doi: 10.15252/embj.2020106003 (PMC7604621; doi:10.15252/embj.2020106003)
Supplement: Supplementary file 9 — Movie EV3 [file EMBJ-39-e106003-s009.zip › 106003_Extended View Movies EV3/106003_Extended View Movie EV3 Legend.docx]

**Movie EV3.** Time-lapse confocal imaging of a tip cell in a *Tg*(*fli1ep:MCP-GFPnls*) zebrafish embryo showing the localisation of MS2 hairpin-tagged *rab13* 3’UTR (T_0_ = 24 hours post fertilisation). Arrowheads indicate non-nuclear localisation of MCP-GFPnls.
