# Supplementary material for: RAB13 mRNA compartmentalisation spatially orients tissue morphogenesis
Source: EMBO J. 2020 Sep 18;39(21):e106003. doi: 10.15252/embj.2020106003 (PMC7604621; doi:10.15252/embj.2020106003)
Supplement: Supplementary file 13 — Source Data for Figure 5 [file EMBJ-39-e106003-s012.pdf]

Blot scans used to generate Fig 5E

(RAB13 blots)

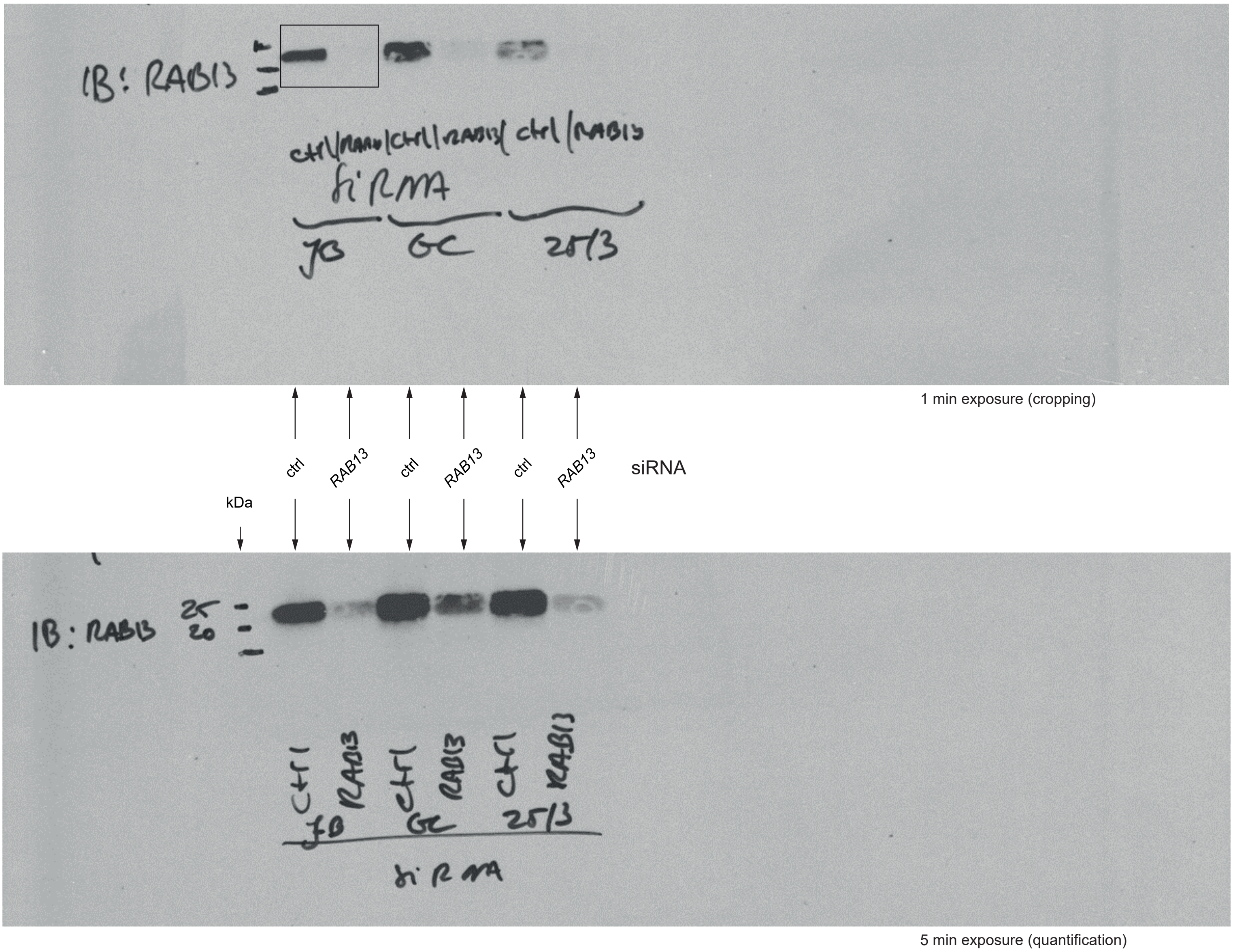

(β-Tubulin blot)

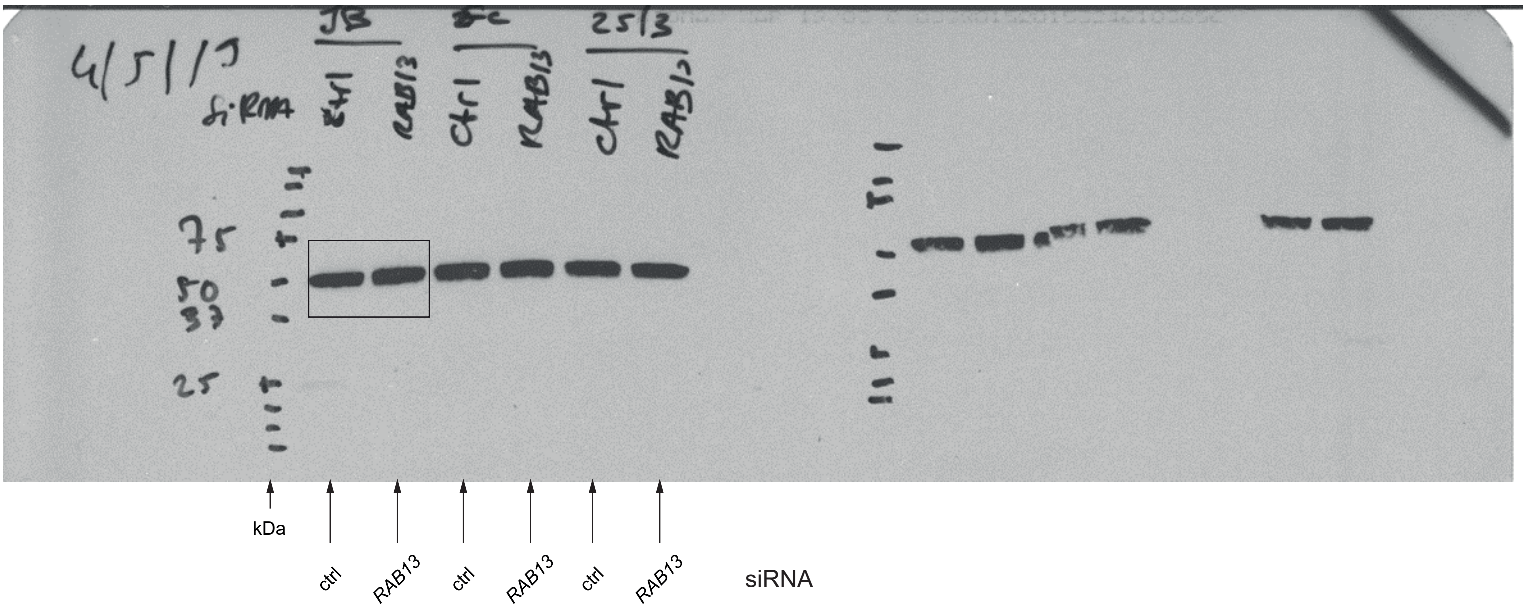

Black boxes indicate areas cropped for the main figures.
